# A fully automated deep learning pipeline for micro-CT-imaging-based densitometry of lung fibrosis murine models

**DOI:** 10.1186/s12931-022-02236-x

**Published:** 2022-11-11

**Authors:** Elena Vincenzi, Alice Fantazzini, Curzio Basso, Annalisa Barla, Francesca Odone, Ludovica Leo, Laura Mecozzi, Martina Mambrini, Erica Ferrini, Nicola Sverzellati, Franco Fabio Stellari

**Affiliations:** 1 Department of Computer Science, Bioengineering, Robotics and Systems Engineering, University of Genoa, Genoa, Italy; 2 Camelot Biomedical System S.R.L, Via Al Ponte Reale 2/20, 16124 Genoa, Italy; 3 Department of Medicine and Surgery, University of Parma, Parma, Italy; 4 Department of Veterinary Science, University of Parma, Parma, Italy; 5 Chiesi Farmaceutici S.P.A, Corporate Pre-Clinical Research and Development, Largo Belloli, 11/A, 43122 Parma, Italy

**Keywords:** Drug discovery, Micro-computed tomography, Deep learning, Segmentation, Preclinical

## Abstract

Idiopathic pulmonary fibrosis, the archetype of pulmonary fibrosis (PF), is a chronic lung disease of a poor prognosis, characterized by progressively worsening of lung function. Although histology is still the gold standard for PF assessment in preclinical practice, histological data typically involve less than 1% of total lung volume and are not amenable to longitudinal studies. A miniaturized version of computed tomography (µCT) has been introduced to radiologically examine lung in preclinical murine models of PF. The linear relationship between X-ray attenuation and tissue density allows lung densitometry on total lung volume. However, the huge density changes caused by PF usually require manual segmentation by trained operators, limiting µCT deployment in preclinical routine. Deep learning approaches have achieved state-of-the-art performance in medical image segmentation. In this work, we propose a fully automated deep learning approach to segment right and left lung on µCT imaging and subsequently derive lung densitometry. Our pipeline first employs a convolutional network (CNN) for pre-processing at low-resolution and then a 2.5D CNN for higher-resolution segmentation, combining computational advantage of 2D and ability to address 3D spatial coherence without compromising accuracy. Finally, lungs are divided into compartments based on air content assessed by density. We validated this pipeline on 72 mice with different grades of PF, achieving a Dice score of 0.967 on test set. Our tests demonstrate that this automated tool allows for rapid and comprehensive analysis of µCT scans of PF murine models, thus laying the ground for its wider exploitation in preclinical settings.

## Introduction

Idiopathic pulmonary fibrosis (IPF), the archetype of pulmonary fibrosis (PF), is a potentially fatal chronic lung disease characterized by a progressively worsening lung function due to the development of fibrous connective tissue as a reparative response to injury [1]. Although two antifibrotic drugs^1^ are now available for the treatment of IPF and other forms of progressive fibrosis, their clinical efficacy is limited and lung transplantation remains the only option to prolong the survival of such patients [2]. The development of new drugs for IPF patients strongly relies on preclinical studies, whose usefulness depends on the ability of animal models to mimic human physiology, disease pathogenesis and response to treatments. Although none of the present pulmonary fibrosis (PF) murine models fully reproduces the features of the human disease, the bleomycin (BLM)-induced PF model is widely used to study disease pathogenesis and evaluate the efficacy of potential new drugs [3]. Current outcomes measured in BLM models of PF involve time-consuming invasive approaches, such as histological scoring that requires animal sacrifice at fixed time-points, thus precluding any longitudinal examination which is essential to fully understand disease development and progression [4].

In clinical practice, computed tomography (CT) plays a key role in the diagnosis and monitoring of lung injuries [5]. It enables a highly reproducible and longitudinal quantification of lung injuries, as recommended by international diagnostic guidelines [6]. Owing to the linear relationship between X-ray attenuation and tissue density, lung densitometry has been demonstrated to be widely feasible, reproducible, and much less time consuming than visual assessment in several lung disorders [7]. Histogram-based measurements refer to Hounsfield Units (HU, also referred as CT-numbers or CT-values) frequency distribution (i.e. physical density distribution). Therefore, information on air levels in specific lung regions can be derived from lung density histograms.

Recently, a miniaturized version of CT (µCT) has been optimized and validated as a tool to assess PF at different time-points in live animals [8]. This imaging modality can be used in the BLM mouse model of PF to understand the pathogenesis of fibrosis by monitoring the air content of specific lung compartments as well as to evaluate the efficacy of new antifibrotic drug candidates.

In the present study, we developed a deep learning approach aimed to localize and segment the left and right lungs in thoracic µCT scans of fibrotic mice, thus allowing an automatic quantitative analysis (lung densitometry). In particular, we propose a fully automated pipeline for left and right lung segmentation in native µCTs. This relies on a first coarse U-Net for pre-processing, followed by other U-Nets for a fine lung segmentation, accounting for spatial coherence. Furthermore, the proposed algorithm automatically subdivides the lungs into functional compartments (i.e., normo-aerated, hypo-aerated, non-aerated, and hyper-inflated) based on µCT voxel density to quantitatively assess changes in specific lung regions while monitoring disease progression.

### Background

Lung segmentation is a prerequisite for any quantitative analysis of CT scans of the chest, including lung densitometry, but manual segmentation is time-consuming and prone to inter-observer and intra-observer variation. Therefore, the development of a fully automated segmentation algorithm to facilitate rapid quantitative analysis of CT images has become an important goal of medical imaging research. Recently, with the advent of increasingly powerful graphics processing units (GPUs), deep learning techniques have shown excellent performance in the field of medical image analysis. Indeed, several deep learning architectures have been proposed as a way to overcome the problems encountered with conventional segmentation algorithms and to handle pulmonary diseases causing lung density changes.^2^To name a few, Gerard et al. [9], Park et al. [10], and Jalali et al. [11] have shown that different types of deep learning approaches can be successfully applied to chest CT imaging.

Besides the different pixel size (mm vs µm), pre-clinical µCT differs from clinical CT in several aspects. The acquisition time of μCT needs to be longer to collect more projections to provide accurate image reconstruction during free-breath scanning. In addition, compared to clinical CT detectors used have a limited dynamic range, typically 12–14 bits (compared with 20 bits in clinical CT). Thus, the integration of advanced automatic algorithms into image processing could pave the way to a fully automated lung densitometric investigation, where fibrosis assessment and evaluation of new candidate drugs will be completely µCT-guided.

Mouse organ segmentation on µCT is challenging even in healthy animals due to the intrinsically low contrast. For this reason, atlas-based approaches were initially proposed instead of intensity-based ones [12]. However, the results obtained with these methods may be affected by multiple factors such as the size of the dataset used to create the atlas, variations in mouse anatomy, and the specific methodology employed for image registration. In recent years, deep learning-based algorithms have also been proposed for organ segmentation in µCT. However, µCT imaging research is mostly performed by academic laboratories, which usually operate on a small-scale in terms of the number of healthy mice employed for experimentation.

Schoppe et al. [13] presented a U-net-based architecture, called AIMOS, that automatically segments major organs, including the lungs, in 2D coronal µCT whole-body sections of healthy mice. The authors added a post-processing step based on an ensemble voting procedure to eliminate outlier predictions. Malimban et al. [14] devised a 2D and 3D U-Net-based nnU-Net architecture for lungs self-construction on µCT of mice thorax, even in the presence of a low contrast. In the work of Sforazzini et al. [15], the developed deep learning model efficiently segmented the lungs on CT images from mice with different degrees of fibrosis. The authors also explored the applicability of a transfer learning approach to achieve lung segmentation using the proposed network from µCT images of healthy mice, but the test set only consisted of four specimens.

Lung densitometry is not addressed in any of the proposed approaches. Birk et al. [16] proposed a deep learning approach to automatically detect the central part of the right and left lung on a µCT stack and perform lung densitometry on these portions of the lung. Many murine models of BLM-induced fibrosis show some limitations in the distribution of the disease across the lobes and more generally across the left and right lungs. Lung densitometry performed on the left and right lung separately may help overcome this limitation of the animal model. However, to the best of our knowledge, no approach proposed so far has been designed for lung segmentation in mouse µCT with significant changes in parenchymal density as those observed in severe PF. In addition, none of the reported approaches integrate an automated densitometric analysis of the entire lung volume or separate left and right lobe volumes.

## Materials and methods

### Dataset

The dataset used in this study has been provided by Chiesi Farmaceutici S.p.A (Parma, Italy)^3^ and consists of Micro Computed Tomography (µCT) scans of female murine models of BLM-induced pulmonary fibrosis.

#### Data acquisition

Pulmonary fibrosis is induced by instillation of BLM (25 µg/mouse for each instillation) on day 0 and 4. Animals were lightly anesthetized with 2.5% isoflurane and bleomycin hydrochloride in saline solution was administered via oropharyngeal aspiration (OA). This protocol was shown to ensure a uniform distribution of fibrotic lesions throughout the lung [17].

On day 7, mice were divided into two groups: healthy (saline) and pathological (BLM).^4^ On days 7, 14, 21 from BLM administration, mice were anesthetized with 2% isoflurane and scanned on a Quantum GX MicroCT PerkinElmer,^5^ Inc. Waltham, MA.

Lungs were scanned 360° with X-ray tube voltage of 90 kV, and current of 88 µA, using ‘high speed’ acquisition mode with a respiratory gating technique. The respiratory gating strategy consists in an intrinsic retrospective two phase gating technique which has been ideally developed for animal models in which measurements can be adversely affected by heart or lung motion.

In particular, once the animal was placed on a scanner bed in supine orientation, the chest was aligned withing the field of view (FOV) using X, Y, and Z axis motorized stage controls in Live mode with the respiratory region of interest (ROI) positioned over the diaphragm. The respiratory signal trace, the respiratory cycle lengths and the respiratory rate were monitored by reading the values inside the Respiratory Synchronization window. Projection images were collected in list-mode over a single continuous gantry rotation, total rotation time of 4 min and 14.688 raw projections are acquired, one per 16 ms (one per 0.024° angle of rotation).

Back-projections associated with different phases of the respiratory cycle are acquired, contrarily to clinical CT that is acquired at the end of a full breath-hold.^6^

**Fig. 1:**
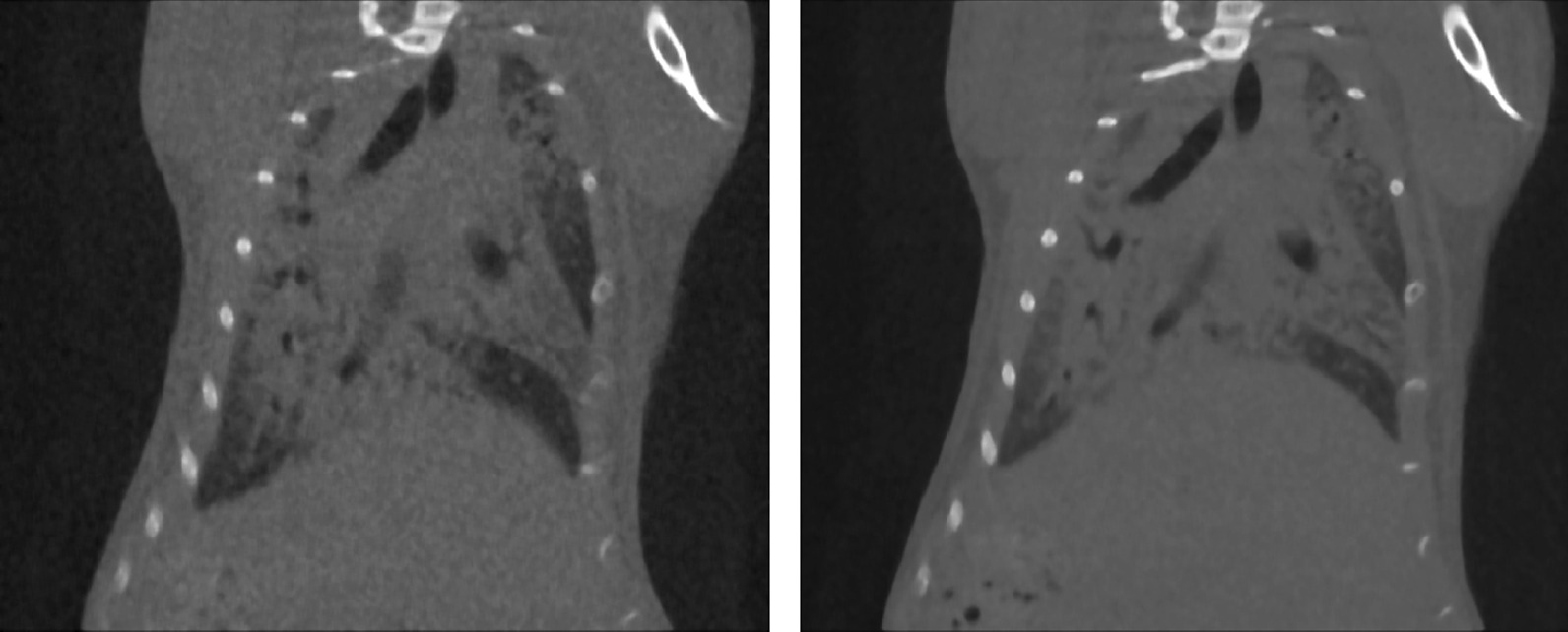
An example of coupled scans: P01 (end-inspiration) scan on the left and P02 (end-expiration) scan on the right. Both scans are shown with window level (WL) = 590 HU and window width (WW) = 4310 HU. In end-inspiration scan, lungs are darker and stretched downward due to air within the lung parenchyma

For the scope of this work only end-inspiratory (P01) and end-expiratory (P02) projections were retrospectively sorted (on average 900 projections for each phase) and automatically reconstructed using a GPU-based filtered back-projection algorithm with a Ram-Lak filter [18]. Therefore, for each acquisition, two stacks of 512 cross-sectional images, resulting in two 3D datasets with 50 µm isotropic reconstructed voxel size and corresponding to the two different phases P01 and P02 of the breathing cycle (end-inspiration and end-expiration, respectively) were produced. Figure 1 shows coupled P01 and P02 scans for a PF mouse.

The system is calibrated monthly with standard phantoms for noise, uniformity, low contrast, and resolution [19]. The µCT scans provided have been carried out from the same device and acquired with the same voxel size (50 × 50 × 50 µm^3^), therefore a voxel size normalization is not required. Indeed, if input scans are acquired by different machines or in different institutions, they are likely to have different spatial resolution. However, to enable networks to learn spatial semantics correctly, it is preferable to choose a fixed voxel size and resample all input scans to that size. Manual segmentations were performed in µCT after the application of a median filter with kernel size 5 × 5 × 5 to remove noise. Following the same procedure, the input scans were filtered in the pre-processing stage. In addition, µCT scanner used in this work provides grey levels images and HU conversion has been implemented to perform the analyses.

The dataset contains 219 µCTs of end-expiration phase (P02) from different longitudinal studies performed in healthy (saline) and pathological animals (BLM). In addition, 83 µCTs of end-inspiration phase (P01) are provided and used to test the generalization capability of the approach.

#### Data annotation

Each µCT volume was segmented semi-automatically by trained operators by means of Analyze software (Analyze 12.0; Copyright 1986–2017, Biomedical Imaging Resource, Mayo Clinic, Rochester, MN),^7^ according to Chiesi Farmaceutici S.p.A. well-established and tested protocol. Since ground truth generation is a time-consuming process, each µCT scan was segmented by a single operator.

Lungs were finely segmented into 167 µCT P02 volumes and 67 µCT P01 volumes (Dataset A). In the remaining 52 P02-phase µCT scans and 20 P01-phase µCT scans, right lung and left lung were finely segmented separately (Dataset B). In addition, heart and airways were coarsely segmented in each µCT volume.

Table 1 shows data partitioning by time point, acquisition phase (end-expiration/end-inspiration) and disease prevalence.

**Table 1:** Data partitioning by time point, acquisition phase (end-expiration P02/end-inspiration P01) and disease prevalence

	Dataset summary											
	0 days		7 days		14 days		21 days		# Scans		Prevalence	
	P02	P01	P02	P01	P02	P01	P02	P01	P02	P01	P02	P01
Dataset A	4	5	41	15	35	21	87	26	167	67	84%	92%
Dataset B	5	5	21	5	5	5	5	21	52	20	69%	60%

#### Data splitting

µCT acquisitions and corresponding segmentations have been divided into three sets (training set, validation set, test set) ensuring that the µCTs acquired for each mouse at different time points were included in the same set. For each training, a fivefold split approach was adopted to evaluate the prediction on each µCT in the dataset and the final model was trained afterwards. Training set and test set are respectively exploited to train and evaluate the network, whereas validation set is used both to prevent overfitting during the training process by early stopping regularization and to define the threshold needed to binarize the coarse probability maps provided by the first network.

### Lung segmentation pipeline

**Fig. 2:**
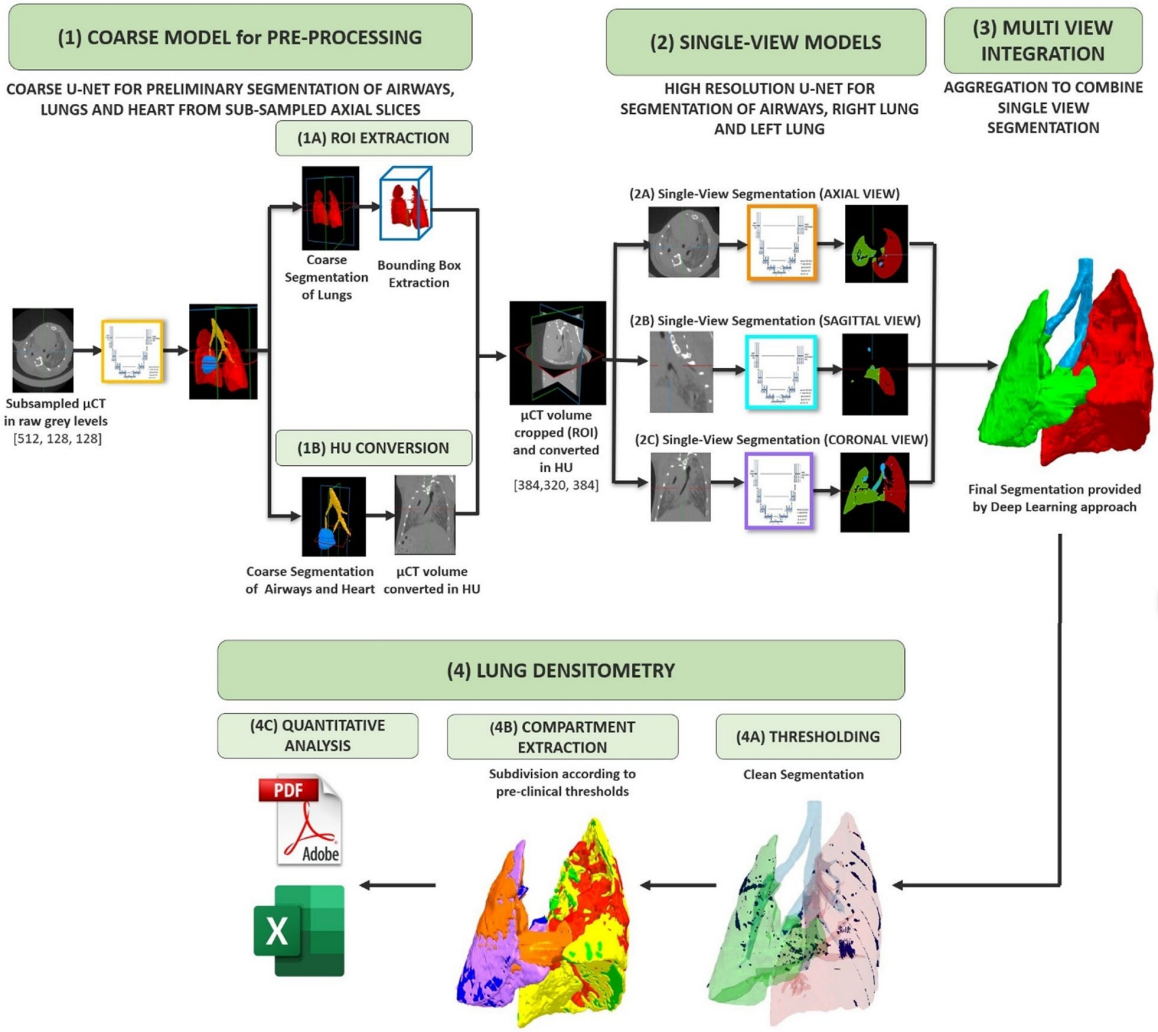
The proposed pipeline for lung segmentation and densitometry. First, a multiclass network is used to identify the lungs, heart, and airways. Heart and airways segmentations are both needed to compute transfer function from raw grey levels into Hounsfield units (HU). Lung segmentation is used to identify the region of interest for the subsequent analysis. Then, three different higher-resolution 2D networks are used to segment the right and left lung on the three views (axial, sagittal, and coronal) of the HU-converted and cropped µCT scans. The result of these three networks is integrated to obtain an accurate and spatially coherent segmentation and thresholded in the [− 1040, + 121] HU range to remove voxels labeled by the network as lungs but whose corresponding value in the µCT scan is outside the appropriate range. Finally, this clean segmentation is compartmentalized based on the corresponding voxel value in µCT according to the thresholds introduced by [20]

The proposed approach for automatic lung segmentation and analysis is described in Fig. 2. A first multiclass 2D U-Net trained on subsampled µCT axial slices (down-sample factor = 4) in raw grey levels is used to extract a preliminary segmentation of lung, airway, and heart from µCT scans and perform pre-processing. Coarse airways and heart segmentations are used to perform conversion from grey levels to HU. Coarse lung segmentation, on the other hand, is used to extract a bounding box in which the lung volume is entirely contained to identify the Region of Interest (ROI) in the higher resolution image. The ROI identified and converted to HU is then processed by three different 2D U-Nets trained separately in the axial, sagittal and coronal planes, obtained by extracting 2D slices along the *x*, *y* and *z* axes of the higher-resolution µCT scan. The predictions provided by the three U-Net planes are then combined to provide a final spatially coherent segmentation of the left and right lungs. Of note, segmentation is considered as a multiclass classification problem, so each pixel in the image is classified as one of the structures of interest or as a background.

#### Lung localization and Hounsfield unit conversion

As mentioned above, µCT scans are not provided in HU. Therefore, as a first step, the transfer function from grey levels to HU is computed. In addition, a Bounding Box (i.e., a cuboid) in which the lung volume is completely contained is identified.

A preliminary multi-class 2D U-Net model is used to segment the airways, lungs, and heart from the axial view. The network is trained and validated on µCT axial slices in raw grey levels resized to 128 × 128 (down-sample factor = 4) to reduce the memory requirement. The probability map provided by the coarse 2D model is binarized with three different thresholds optimized on the validation set to obtain the preliminary segmentations of the airways, lungs, and heart. The resulting coarse segmentations of the airways and heart are used to extract the average grey level values of these two structures. The transfer function from raw grey levels (GL) to Hounsfield unit (HU) is a linear function, computed simply by imposing that the average grey level values of airways and heart are − 1000 and + 50, respectively. In our settings, the mean values of the raw grey levels for airways and heart are 801.8 and 2250.19, respectively. The derived transfer function is simply applied to the μCT in raw grey levels at full resolution to obtain the μCT in HU. This step is critical for calculating lung densitometry because it introduces a linear relationship between the µCT image unit and tissue density. The resulting coarse segmentation of the lungs, on the other hand, is used to extract a bounding box of the lung mask. A cuboid of dimension 384 × 320 × 384 centred in lungs is used to crop the converted µCT image at higher resolution. This step is performed to crop the µCT and its segmentation and thus retain only the information useful for segmentation, excluding part of the background to speed up the next steps.

#### Single-view segmentation

Each µCT scan is analyzed in axial, sagittal, and coronal 2D views. The scans are converted to HU and cropped with the cuboid based on the information calculated in the first step from the coarse segmentations. The cropped and converted scans are down sampled (down-sampling factor = 2) from the original µCT scans to meet memory requirements.

Initially, a deep learning model based on the same U-Net architecture is trained separately to segment the lungs on each orthogonal view. Subsequently, we used transfer learning to obtain the models able to segment right and left lung separately: in particular, the decoding path of the three previously obtained models was retrained with the data for which segmentation into right/left lung was available, while the encoder path was frozen during the network retraining.

This step was necessary because of the low number of data labeled with right and left lung. In this way, we trained the model to identify the entire lung morphology and then to discriminate between right and left lung.

The outputs provided by the single plane networks are 2D probability maps where each intensity value represents the probability that a given pixel is right lung, left lung, or background. Analogously to coarse segmentation, the single-view label map is obtained by binarizing the prediction map with two thresholds optimized on the validation set.

#### Multi-view aggregation

The aggregation stage is intended to regularize the voxel prediction by considering the spatial information from the three orthogonal views. Each single-view network makes a prediction for the voxels in the µCT scan and the corresponding label maps are generated. The Majority Voting integration approach was implemented to combine single-view segmentation. According to the approach suggested by Zhou et al. [20], the predicted label (right lung, left lung or background) is assigned to the voxel following the majority voting rule among the predictions of the single-view networks.

### Lung densitometry

Mecozzi et al. [20] proposed densitometric cut-off values to identify lung abnormalities such as fibrosis and emphysema in animal models. The ranges introduced are [− 121, + 121] HU for Non-Aerated, [− 435, − 121] HU for Hypo-Aerated, [− 860, − 435] HU for Normo-Aerated, [− 1040, − 860] HU for Hyper-Inflated.

The segmentation obtained by deep learning is automatically subdivided into lung compartments according to the thresholds proposed by Mecozzi et al., and quantitative analyses for assessing disease progression in different subjects are performed. Figure 1 shows proposed pipeline. First, the final segmentation provided by the deep learning model is thresholded in the range [− 1040, + 121] HU. This step is performed to remove voxels labeled as lungs by the network, but whose corresponding value in the µCT scan is outside the appropriate range. Then, this *clean* segmentation is compartmentalized based on the corresponding voxel value in µCT (i.e. the aeration) according to the thresholds introduced and quantitative analyses are performed.

### Metrics and data analysis

The segmentations and conversions in HU obtained with the proposed pipeline are compared to ground truth annotations using different criteria:

- *Dice score (DSC) of the segmentation network*: used to evaluate the performance of the network as an overlap measure between the predicted and the ground truth segmentations. The Dice coefficient between two binary segmentations is defined as follow:
  $$DSC=\frac{2\left|GT\cap Pred\right|}{\left|GT\right|+\left|Pred\right|}$$
  where $GT$ is the ground truth volume and $Pred$ is the automatically segmented volume.
- *Absolute percent error of volume*: represents the absolute value of the difference in volume between the volume calculated from the predicted segmentation and volume calculated from the ground truth segmentation divided by the volume calculated from ground truth segmentation: $\frac{\left|Vo{l}_{Pred}-Vo{l}_{GT}\right|}{Vo{l}_{GT}}$
  $Vol$ is calculated as the number of non-zero voxels in lung segmentation multiplied by the volume of the single voxel 50^3^ µm^3^ = 12.5 10^–5^ mm^3^).
- *Percent absolute error of compartment:* is the absolute value of the difference between the percentage of a certain compartment on total volume calculated from the predicted segmentation and that calculated from the ground truth segmentation.
  $$\left|\%Vo{l}_{Pred}^{comp}-\%Vo{l}_{GT}^{comp}\right|$$

This metric represents a more complete evaluation because it involves both the conversion to HU and the predicted segmentation.

## Experiments and results

### Experimental settings

The experiments were performed on 219 scans acquired at the Chiesi Farmaceutici S.p.A Laboratory in Parma (Italy). Additional 87 scans were used for testing. Both training, validation and testing were performed on a NVIDIA Tesla K40c graphic card with CUDA compute capability = 3.5 and a NVIDIA Titan Xp with CUDA compute capability = 6.1, under Ubuntu operating system. The deep learning models were implemented in Python using Keras framework based on Tensorflow with GPU support. These models, developed for Chiesi Farmaceutici S.p.A, are routinely used to perform predictions in new µCTs using an NVIDIA Tesla P40 graphics card with CUDA = 6.1 computational capability, under Windows operating system.

For model trainings, categorical cross-entropy was used as a loss function in the coarse multi-class model and the final single-view multi-class models. Binary cross entropy was used for the intermediate single-view models trained to segment the lungs jointly. The Adam optimizer with learning rate = 0.0001 was adopted to optimize the network parameters. In each iteration, a mini-batch containing 24 and 4 slices randomly sampled from the training set was provided to the coarse U-Net and single view U-Net, respectively. The training process was stopped using early stopping criteria, with patience set to 10 epochs.

A data augmentation procedure was adopted: images and masks in the mini batch were modified on the fly during the training process with random rotations, shifts, and zoom factors to augment training and validation sets. The transformation parameters were extracted randomly from a uniform distribution range of maximum variation of [− 5°,5°] for rotation, [− 5%, + 5%] for shifting and [− 15%, + 15%] for zooming.

Only P02 scans were used for model training and validation because densitometric analyses are typically performed on end expiratory (P02) scans. This allows to avoid any possible bias caused by lung-entrapped respiratory air as in end-inspiratory (P01) scans where the specimen breathing capacity may affect image attenuation.

For both coarse and single-view models, fivefold split approach was adopted to evaluate the prediction on each µCT in the dataset. Specifically, 5 different models were initially trained on 5 different splits of the dataset. A final model was then trained by setting the number of epochs and the binarization thresholds as medians of the parameters obtained in the splits to train the model on as many data as possible. Approximately 5% of the data was used as a control test set. During the k-fold splitting process, models were tested on end-expiration (P02) µCTs volumes only. For single-view models, the above procedure was initially applied to *Dataset A*. This dataset was comprised of 167 µCTs and allowed end-to-end learning of lung segmentation. Then, the final model thus obtained was used as a starting point to perform the same procedure (k-fold splitting and end-to-end training) a second time using *Dataset B*. This dataset was comprised of 52 µCTs and allowed learning of right and left lung segmentation by transfer learning from models that segmented these two structures together.

Table 2 shows the number of training, validation, and test sets for each step. The proposed pipeline was further tested on the end-inspiration µCTs volumes (P01).

**Table 2:** Data Splitting in training, validation and test set for Dataset A and Dataset B in each split and in final training

			Data splitting			
	# train		# validation		# test	
	*A*	*B*	*A*	*B*	*A*	*B*
Split 1	144	34	29	8	36	10
Split 2	93	32	36	12	30	8
Split 3	108	34	32	6	33	12
Split 4	107	32	31	10	37	10
Split 5	115	36	30	8	31	8
Final	195	46	0	0	14	6

### Lung localization and Hounsfield unit conversion

The first U-Net has two different purposes: (1) coarsely identify the lungs to extract a bounding box through which cut µCT scans; (2) coarsely locate heart and airways to derive the transfer function from raw grey levels to HU with which to convert the µCT volume.

In the first case, our main interest was the quality of segmentation, which, therefore, was evaluated in terms of a DSC. The coarse segmentations of the lungs predicted by the preliminary model featured an average DSC of 0.933 ± 0.084 on the µCT volumes.

In the second case, however, our main interest was not segmentation but, rather, the average grey value of the segmented structures. In addition, the ground truth segmentations related to the heart and airways are not accurate enough so the DSC would provide unreliable evaluations. Therefore, coarse segmentation of the airways and heart was evaluated by comparing the mean grey values and the slope and intercept of the transfer function derived automatically, via neural network, with those derived manually in the Chiesi Farmaceutici Laboratory. Lung densitometry related to ground truth segmentation derived from automatically converted µCT scans was also compared with the original, manually converted µCT scans generated in the Chiesi Laboratory.

Lung compartments were calculated based on previously tested thresholds [20] using manual lung segmentation (ground truth) to construct the mask. Table 3 shows the errors obtained regarding raw grey values detected, transfer function parameters and compartmental volume derived from densitometry. More specifically, in the first part, we report the absolute percentage error between the average grey level values derived from manual and network coarse segmentation for both airways and heart. In the second part, the absolute percentage error relative to slope and intercept of the transfer function obtained from manual and network coarse segmentation is reported. For the last evaluation, the scan was converted by two different transfer functions: one obtained from airway and heart manual segmentations and the other one from automatic segmentations. We have computed the percent absolute error (defined in Sect. 2.4) in lung densitometry of the two different conversions, using the manual lung segmentation as mask. In this way, we have verified that using a transfer function based on automatic segmentation results in a lung densitometry very similar to the one obtained using manual segmentation.

**Table 3:** A summary of the evaluations performed to assess the goodness of automatic HU conversion

HU conversion evaluations	
Raw Grey levels detected	
Structure	Absolute percentage error
Airways	4.3% ± 1.9%
Heart	0.1% ± 0.1%

Transfer function	
Parameter	Absolute percentage error
Slope	3.6% ± 0.2%
Intercept	3.4% ± 0.3%

Lung densitometry	
Compartmental volume	Percent absolute error
Normo-aerated volume	0.5% ± 5.6%
Hypo-aerated volume	2.9% ± 4.8%
Non-aerated volume	0.4% ± 6.7%

The final preliminary model was then trained by keeping a small control test set. Table 4 shows the data for this training procedure and the performance on the control test set.

**Table 4:** A summary of data employed to perform coarse axial model together with information on the training process

Coarse axial model summary							
	Number of train µCT	Number of test µCT	Image dimension	Number of epochs	Training time [h]	Binarization threshold	Lung DSC on test set
Coarse	195 (99,840 2D slices)	14 (7168 2D slices)	(128, 128)	28	02:35:37	[0.45, 0.45, 0.45]	0.939 ± 0.040

### Single-view lung segmentation

Axial, sagittal, and coronal U-Nets were separately trained on the converted µCT cropped around the ROI using *Dataset A*. Segmentations predicted by axial, sagittal, and coronal single-view models achieve mean DSC of 0.943 ± 0.034, 0.938 ± 0.055, 0.943 ± 0.035, respectively, over the splits. Then, the final axial, sagittal, and coronal models were trained, keeping a small control test set. Table 5 shows the data employed in this training and the performance on the control test set for the three views.

**Table 5:** A summary of data employed to perform single-view segmentation model together with information on the training process

Single-view models summary on single class (lungs)							
	Number of train µCT	Number of test µCT	Image dimension	Number of epochs	Training time [h]	Binarization threshold	Lung DSC on test set
Axial	195 (74,880 2D slices)	14 (3376 2D slices)	(192, 120)	4	05:49:37	0.55	0.943 ± 0.038
Sagittal	195 (37,440 2D slices)	14 (2688 2D slices)	(192, 192)	10	06:52:29	0.55	0.934 ± 0.055
Coronal	195 (31,200 2D slices)	14 (2240 2D slices)	(192, 120)	9	06:37:13	0.5	0.943 ± 0.037

These models are able to segment the lungs together but not to segment separately right and left lungs. Therefore, the decoding path of the single-view models was re-trained on µCT and related segmentation maps with the distinction between right and left lungs (*Dataset B*). Axial segmentations of right and left lung predicted by the model yielded a mean DSC of 0.964 ± 0.021 and 0.954 ± 0.027, respectively, over the splits. Sagittal segmentations of right and left lung featured a mean DSC of 0.948 ± 0.033 and 0.940 ± 0.029, respectively, over the splits. Coronal segmentations of right and left lung record a mean DSC of 0.965 ± 0.017 and 0.957 ± 0.020, respectively, over the splits.

Then, the final axial, sagittal, and coronal models for segment left and right lungs were trained, keeping a small control test set. Table shows the data employed in this training and the performance on the test set for the three views.

### Multi-view aggregation

Multi-view aggregation is a crucial step in the proposed pipeline. A majority Voting approach was implemented to integrate axial, sagittal, and coronal predictions into a final segmentation. In addition, by combining the left and right lung segmentations and binarizing the resulting probability map, total segmentation was achieved.

**Fig. 3:**
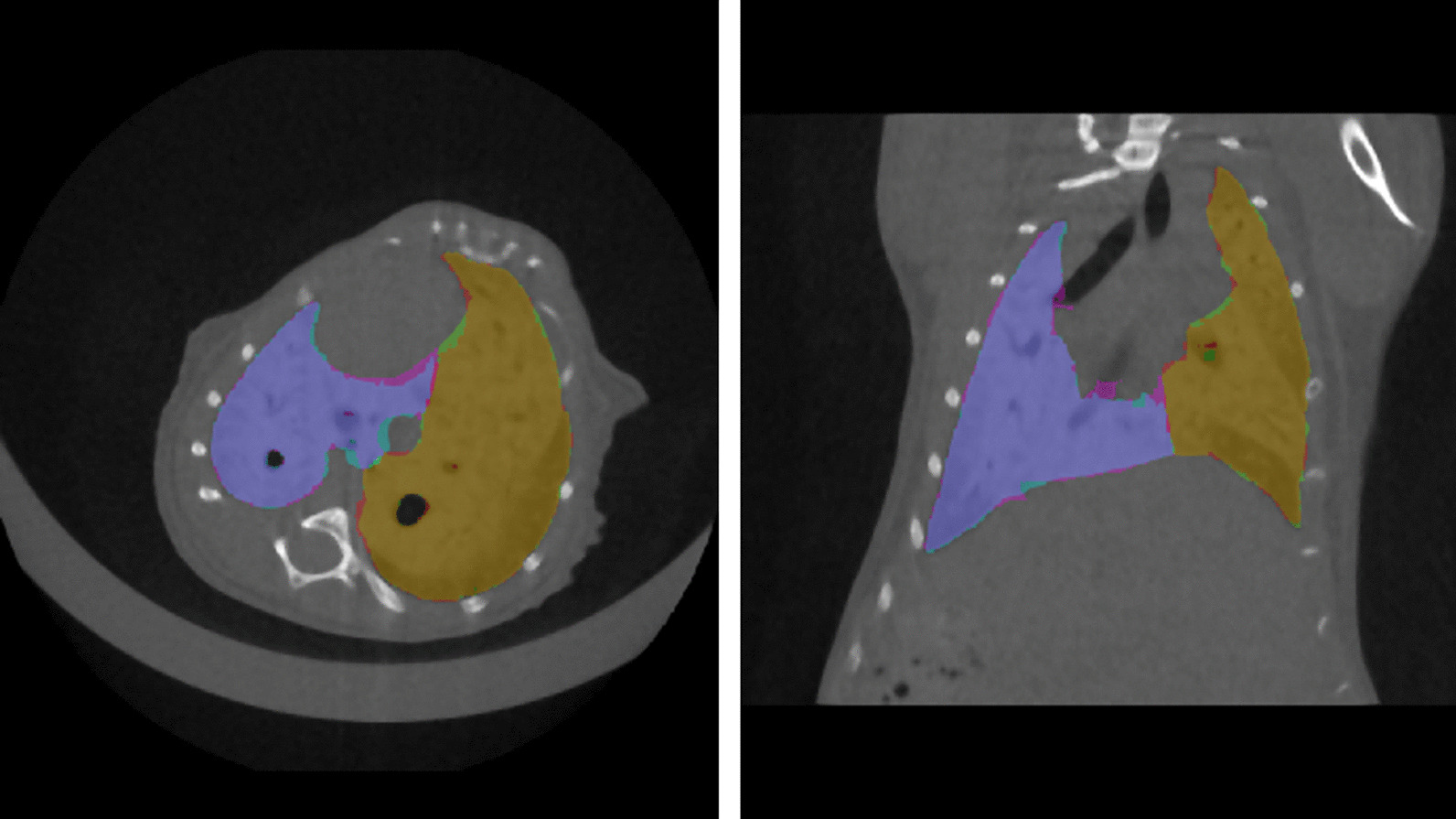
Ground truth and predicted segmentations are overlaid on the corresponding µCT belonging to the test set. The segmentation ground-truth segmentation is shown in magenta for the left lung and green for the right lung, while automatic segmentations are shown in cyan for the left lung and red for the right lung. The overlap of the two segmentations is then shown in purple for the left lung and in orange for the right lung. Both scans are shown with window level (WL) = 590 HU and window width (WW) = 4310 HU

The results obtained with multi-view aggregation were compared with those generated by single-view segmentations to evaluate the impact of the proposed approach on the test set. As shown in Table 6, the combination of orthogonal segmentations led to better results compared to those of single-view segmentations. The models were only evaluated on *Dataset B* because transfer learning was performed on the models trained on *Dataset A*. Figure 3 shows the ground truth and predicted segmentations overlaid on a µCT scan with a high degree of fibrosis.

**Table 6:** A summary of data employed to perform single-view right/left segmentation model together with information on the training process

Single-view models summary on multi-class (left lung/right lung)								
	Number of train µCT	Number of test µCT	Image dimension	Number of epochs	Training time [h]	Binarization threshold	Left lung DSC on test set	Right lung DSC on test set
Axial	46 (17,664 2D slices)	6 (820 2D slices)	(192, 120)	6	02:19:12	[0.45, 0.45]	0.952 ± 0.021	0.963 ± 0.043
Sagittal	46 (39,168 2D slices)	6 (1152 2D slices)	(192, 192)	9	02:53:39	[0.40, 0.40]	0.940 ± 0.031	0.950 ± 0.042
Coronal	46 (32,640 2D slices)	6 (960 2D slices)	(192, 120)	11	02:27:39	[0.40, 0.40]	0.957 ± 0.028	0.964 ± 0.027

**Fig. 4:**
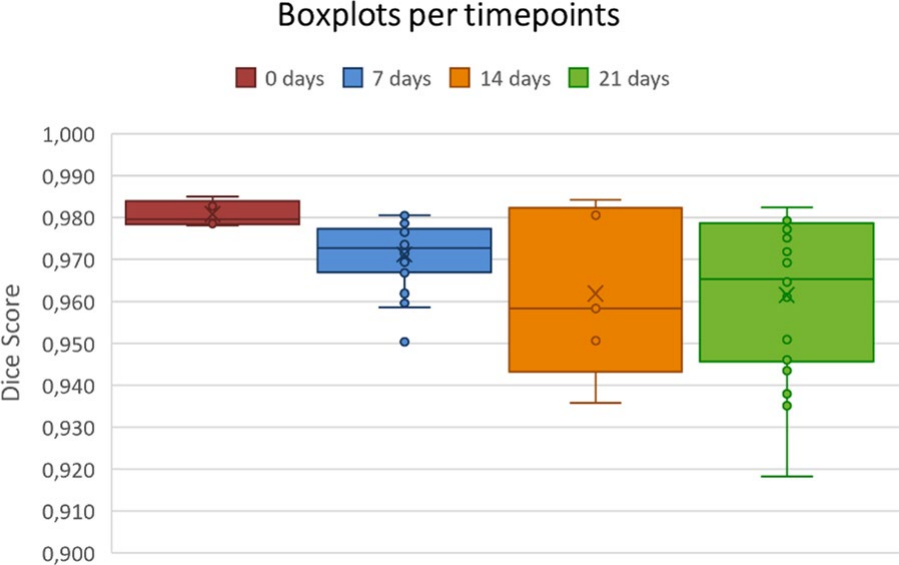
Boxplots related to pipeline performance on the left and right lung segmentation divided by timepoint

To corroborate the quality of the models, their performances were divided according to the corresponding time-points and relative DSC values were calculated (see boxplots in Fig. 4). Furthermore, to test the generalization capability of the models, the optimized pipeline was also tested on P01 scans. The resulting DSC values were 0.926 ± 0.077 on the left lung, 0.950 ± 0.044 on the right lung and 0.949 ± 0.38 on both lungs.

To eliminate out-of-band voxels, the neural network-generated segmentation was thresholded in the range [− 1040, + 121] HU. The volume calculated by ground truth segmentation was compared with the volume yielded by automatic segmentation. The absolute percent error thus achieved was + 2.6 ± 1.9% on the left lung, + 1.9 ± 1.7% on the right lung and + 2.2 ± 1.6% on both lungs.

### Lung densitometry

**Fig. 5:**
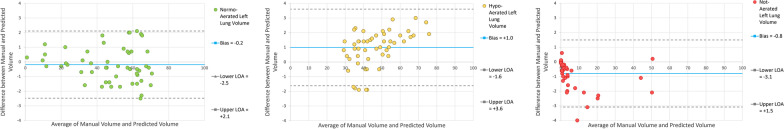
Bland Altman plots of normo-aerated (left), hypo-aerated (center) non-aerated (right) compartment of the left lung which is usually the most affected by the disease. The corresponding Bland Altman plots relating to the right lung are not shown for brevity but show similar results. In all graphs, almost all data are within the confidence interval [Lower LoA, Upper LoA]

**Fig. 6:**
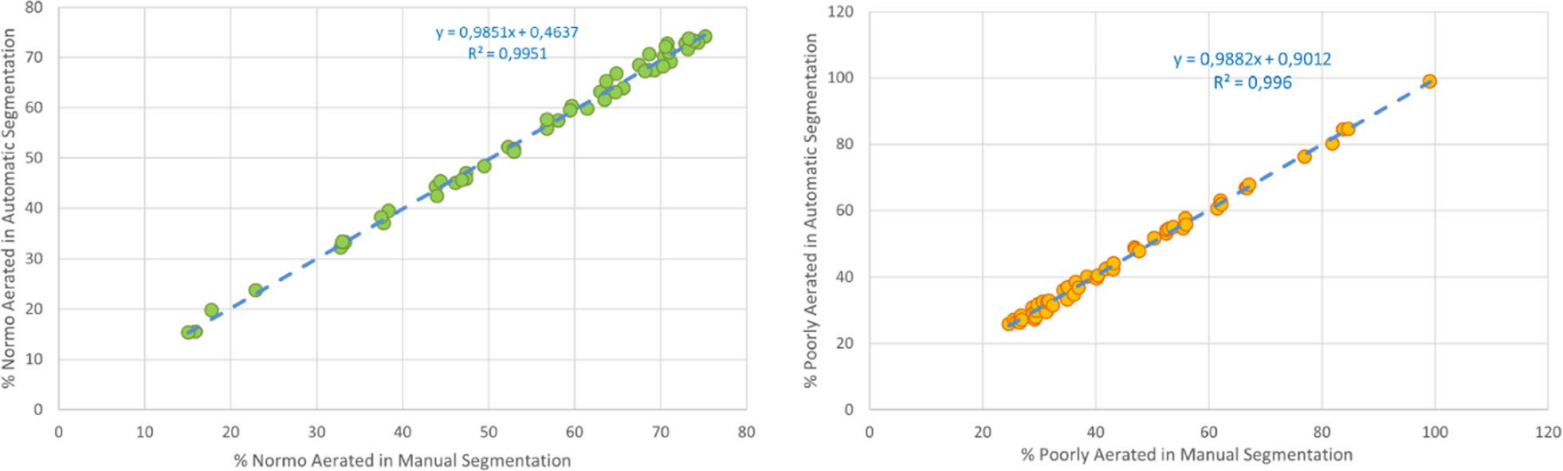
Scatter plots of manual compartment volume (derived from ground truth) vs. predicted compartment volume (derived from pipeline prediction): on the left side, normo-aerated compartment and, on the right side, poorly-aerated (hypo-aerated + non-aerated) compartment. In both cases, the trend line shows good significant correlation between the two measures with a R^2^ = 0.99

The clean segmentation was compartmentalized based on the corresponding voxel values in µCT scans (i.e., aeration) according to pre-defined thresholds [20]. Table 7 shows the difference in percent points between a specific compartment detected in manual segmentation (ground truth) and in automatic neural network segmentation. As shown by the Bland Altman plots of left-lung compartments in Fig. 5, nearly all data are within the confidence intervals highlighted by the dashed grey lines. We also compared the percentage of normo-aerated compartments and poorly-aerated compartments (hypo-aerated + non-aerated) derived from manual volume and automatic total volume analyses. As shown by the scatter plots in Fig. 6, the trend lines indicate a significant correlation between manual and automatic measurements in both compartments (*R*^*2*^ = *0.99*).

**Table 7:** Evaluation of percent absolute error of compartment in right, left and total lung volume

Lung densitometry			
	Left lung	Right lung	Both lung
Normo	− 0.6% (− 2.0%, + 1.6%)	− 0.3% (− 1.7%, + 1.9%)	− 0.5% (− 1.9%, + 1.8%)
Hypo	+ 1.3% (− 1.3%, + 2.9%)	+ 1.4% (− 1.8%, + 2.8%)	+ 1.5% (− 1.5%, + 2.3%)
Non	− 0.1% (− 3.5%, + 0.1%)	− 0.4% (− 2.8%, + 0.1%)	− 0.2% (− 2.8%., + 0.1%)
Hyper	+ 0.0% (− 0.1%, + 0.1%)	+ 0.0% (− 0.1%, + 0.1%)	+ 0.0% (− 0.1%, + 0.1%)

## Discussion

In this study, we present a deep learning approach for spatially coherent lung segmentation and lung densitometry based on whole lung volumetric µCT data. To the best of our knowledge, this is the first approach applied on µCT scans for lung segmentation in fibrotic mice.

Several pulmonary segmentation approaches have previously been proposed for clinical CT analysis of IPF patients [9–11] but the preclinical setting has received considerably less attention, except for a few reports dealing with healthy animals [13–15]. These approaches, however, do not work in the presence of the marked lung parenchyma alterations associated to pulmonary diseases. Indeed, such alterations significantly affect the contrast of the lungs in µCT, making it difficult to distinguish them from the surrounding soft tissue. Our dataset, conversely, includes multiple independent µCT scans from healthy and BLM animals with different degrees of fibrosis.

Our segmentation pipeline is based on the U-Net architecture proposed by Ronneberger et al. [21]. This procedure can perform efficient image segmentation using a limited number of labeled training images and it is thus considered the gold standard in medical segmentation [22].

The first step of the proposed pipeline includes automatic coarse identification of the lungs from µCT scans. This step is performed to retain only the information needed to segment the lungs, excluding some of the background and speeding up subsequent steps. This approach has been widely adopted in medical image segmentation, especially when the structures of interest are small compared to the image size. In the work proposed by Fantazzini et al. [23] for aorta segmentation in angio-CT images, a first 2D U-Net was exploited to extract the ROI from subsampled CT images, then a second 2D U-Net performs a finer segmentation on higher-resolution cropped CTs. A similar approach, based on a 3D U-Net, was proposed by Jia et al. [24] for segmentation of the left atrium in MR images.

In our pipeline, we extend these approaches by using an initial 2D U-Net to simultaneously segment airways and heart in order to obtain the information required for HU conversion in a single step without slowing down the overall pipeline. In this way, our HU conversion approach allows us to automatically examine all those scans for which a reference (e.g., a phantom) is not available for conversion. Future work will address the possibility of designing a procedure for HU conversion independently from segmentation of other structures, in order to further speed up the pipeline and eliminate its dependence on the quality of segmentations.

We preferred a multi-view orthogonal integration approach of 2D CNN rather over a single 3D CNN for finer lung segmentation.

3D CNNs allow to extract more discriminative information than 2D CNNs because the kernels learn volumes rather than sections, and this is useful for segmenting large organs (such as lungs). Kleesiek et al. [25] used CNN 3D to extract brain boundaries, with an improvement of approximately 6% compared to other methods. However, 3D CNNs require a significantly higher number of parameters compared to 2D CNNs and, as a result, are much more resources- and time-demanding, usually needing image subsampling. Moreover, 3D CNNs often require very large data set to enable end-to-end learning and memory requirements are usually very high [25]. On the other hand, the multi-view integration of CNN 2D (the so-called 2.5D approach) is a widely used alternative because it offers a good balance between overall performance and computational efforts [23, 25]. In particular, it is possible to preserve an high image resolution, which is one of the most important issues, especially for small structures or low contrast images such as µCT. With our architecture, a 2D single-view Unet network has 34,535,875 parameters while a 3D Unet network has 103,546,435 parameters. The 2.5D approach requires 3 single-view Unet networks and therefore 3 × 34,535,875 = 103,607,625 parameters. Thus, the number of parameters in our 2.5D approach is slightly larger than that required by a 3D approach. However, in the 2.5D approach the three nets are trained independently, so the number of parameters involved for each single-view training is that of a 2D Unet. On the contrary, in 3D networks all the parameters are involved simultaneously and therefore the computational resources required are much greater. The huge memory requirement is often addressed by limiting the complexity of the model. Moreover, because in our 2.5D approach each µCT volume is decomposed into axial, sagittal and coronal 2D slice stacks, the single-view 2D networks can be trained with a large amount of data. This advantage is not exploited with a 3D network, that processes the whole µCT scans, thus requiring more training data than a 2D network. In addition, 2.5D approaches often return results with comparable accuracy to 3D approaches but with lower computational efforts.

A thresholding step was included after segmentation to remove voxels labeled by the network as lungs but whose corresponding value in the µCT scan is outside the appropriate range [20]. Indeed, it is possible that small portions of ribs or tissue areas of the airways are erroneously included in the lung segmentation. This is because models learn from manual segmentations in a supervised manner, and manual segmentation may contain the above-mentioned errors. To limit this effect, it would be necessary to collect multi-user segmentations for each individual µCT in the training set, thus making the model more robust and limiting errors in areas that are difficult to segment. In our dataset, however, out-of-range voxels excluded from the predicted segmentations however are typically less than 1% of the total lung volume.

The final step in the proposed pipeline involves the computation of lung densitometry.

Considering only a specific portion of the lung [16], even a central one, might be reductive because the most relevant changes in lung parenchyma density are usually localized to peripheral regions such as the accessory lobe or the right apical lobe. In our work, instead, densitometry was performed on the the whole lung volume, taking into account all areas of the lungs and, consequently, more meaningful parameters were obtained. This approach also allows to determine the fractional content (percentage) of singular compartments relative to the total volume of the lungs to monitor their variation during disease development and/or treatment with candidate new drugs.

## Conclusion

In clinical settings, chest CT is widely used for diagnostic purposes and plays a crucial role in patient management [5]. A large community of radiologists is increasingly committed to the creation and distribution of new CT-based diagnostic tools and algorithms [9–11, 26]. In the pre-clinical setting, conversely, the deployment of µCT and automatization approaches are much less common. Imaging research based on µCT is mostly performed in academic centers and is usually limited to relatively small numbers of healthy mice. This contrasts with the needs of fibrosis drug discovery, where µCT imaging has to be applied to a large number of mice and to provide useful results in a relatively short time in order to support the efforts of pharmaceutical companies, aimed at quickly identifying the most promising drug candidates for clinical development.

The key role played by µCT imaging in this drug discovery process has been corroborated by the results of several studies documenting the correlation between in-vivo µCT and ex-vivo histological analysis [8, 20].

In addition, µCT enables longitudinal studies, which in addition to a more detailed monitoring of disease progression, allows a sizable scaling down of the number of required animals. In this work, we presented a fully automated time-saving tool that enables densitometric analysis of the entire lung in murine µCT scans even in the presence of large changes in tissue density. Areas of dense tissue are particularly important when studying the mechanisms involved in pulmonary fibrosis as well as the effects of new potential antifibrotic strategies in murine models.

However, available semi-automated software fails to detect these regions and in order to avoid potential artifacts (and associated confounding effects) segmentations must be corrected manually by trained operators. Considering that drug discovery experiments may require the analysis of up to 1000 scans/year, our approach can significantly reduce the experimental workload. To better substantiate this time-saving, we note that, on average, our pipeline took about 81 ± 15 s per scan (48 ± 9 s per segmentation, 33 ± 6 s per densitometry analysis) compared to the > 40 min required by a complete manual analysis performed under severe pathological conditions (coarse segmentation of airways and heart, accurate segmentation of right and left lungs, conversion to HU and lung densitometry). Our automated pipeline is being used successfully for drug discovery experiments and independently validated at Chiesi Farmaceutici S.p.A.

We also anticipate that the proposed pipeline, although developed in mice, could be extended and adapted to other animal models with different lung anatomies such as rabbits or rats. The only requirement to set-up a transfer learning procedure and create dedicated models for these alternative animal models, would be the availability of a sufficient number of µCT volumes and corresponding manual segmentations.

## Data Availability

The datasets used during the current study are not available from the corresponding author.

## Abbreviations

IPF: Idiopathic pulmonary fibrosis
PF: Pulmonary fibrosis
µCT: Micro-computed tomography
HU: Hounsfield unit
BLM: Bleomycin
GPU: Graphics processing unit
ROI: Region of interest
GT: Ground truth
CNN: Convolutional neural networks
DSC: Dice score

## Footnotes

1 Nintedanib and pirfenidone.

2 Idiopatic pulmonary fibrosis (IPF, chronic obstructive pulmonary disease (COPD), Coronavirus Disease 2019 (COVID-19), Interstinal lung abnormality (ILA).

3 https://www.chiesi.com/.

4 All animal experiments described herein were authorized by the official competent authority and approved by the intramural animal-welfare body (AWB) of Chiesi Farmaceutici and authorized by the Italian Ministry of Health (protocol number: 449/2016-PR). All procedures were conducted in compliance with the European Directive 2010/63 UE, Italian D.Lgs 26/2014, the revised “Guide for the Care and Use of Laboratory Animals” and with the Animal Research: Reporting of In Vivo Experiments (ARRIVE) guidelines.

5 https://www.perkinelmer.com/it/.

6 On average, the acquisition time is 3 s, but it may vary from system to system, depending on the sensitivity of the detector and dynamic range differences.

7 https://www.analyzedirect.com.
